# Role of the NaHCO_3_ Transporter MpsABC in the NaHCO_3_–β-Lactam-Responsive Phenotype in Methicillin-Resistant Staphylococcus aureus

**DOI:** 10.1128/spectrum.00141-23

**Published:** 2023-04-27

**Authors:** Sook-Ha Fan, Richard A. Proctor, Selvi C. Ersoy, Adhar C. Manna, Ambrose L. Cheung, Friedrich Götz, Henry F. Chambers, Arnold S. Bayer

**Affiliations:** a The Lundquist Institute, Torrance, California, USA; b Department of Medicine, University of Wisconsin School of Medicine and Public Health, Madison, Wisconsin, USA; c Department of Medical Microbiology/Immunology, University of Wisconsin School of Medicine and Public Health, Madison, Wisconsin, USA; d Department of Microbiology & Immunology, Geisel School of Medicine at Dartmouth, Hanover, New Hampshire, USA; e Microbial Genetics, Interfaculty Institute of Microbiology and Infection Medicine Tübingen, University of Tübingen, Germany; f UCSF School of Medicine, San Francisco, California, USA; g Geffen School of Medicine at UCLA, Los Angeles, California, USA; University of Pittsburgh

**Keywords:** methicillin-resistant *Staphylococcus aureus*, MRSA, sodium bicarbonate, CO_2_, β-lactams, bicarbonate transporter, membrane potential-generating system ABC, MpsABC

## Abstract

Methicillin-resistant Staphylococcus aureus (MRSA) infections are an increasing concern due to their intrinsic resistance to most standard-of-care β-lactam antibiotics. Recent studies of clinical isolates have documented a novel phenotype, termed NaHCO_3_ responsiveness, in which a substantial proportion of MRSA strains exhibit enhanced susceptibility to β-lactams such as cefazolin and oxacillin in the presence of NaHCO_3_. A bicarbonate transporter, MpsAB (membrane potential-generating system), was recently found in S. aureus, where it plays a role in concentrating NaHCO_3_ for anaplerotic pathways. Here, we investigated the role of MpsAB in mediating the NaHCO_3_ responsiveness phenotype. Radiolabeled NaH^14^CO_3_ uptake profiling revealed significantly higher accumulation in NaHCO_3_-responsive vs nonresponsive MRSA strains when grown in ambient air. In contrast, under 5% CO_2_ conditions, NaHCO_3_-responsive (but not nonresponsive) strains exhibited repressed uptake. Oxacillin MICs were measured in four prototype strains and their *mpsABC* deletion mutants in the presence of NaHCO_3_ supplementation under 5% CO_2_ conditions. NaHCO_3_-mediated reductions in oxacillin MICs were observed in the responsive parental strains but not in *mpsABC* deletion mutants. No significant impact on oxacillin MICs was observed in the nonresponsive strains under the same conditions. Transcriptional and translational studies were carried out using both quantitative reverse transcription-PCR (qRT-PCR) and *mpsA*-green fluorescent protein (GFP) fusion constructs; these investigations showed that *mpsA* expression and translation were significantly upregulated during mid-exponential-phase growth in oxacillin-NaHCO_3_-supplemented medium in responsive versus nonresponsive strains. Taken together, these data show that the NaHCO_3_ transporter MpsABC is a key contributor to the NaHCO_3_–β-lactam responsiveness phenotype in MRSA.

**IMPORTANCE** MRSA infections are increasingly difficult to treat, due in part to their resistance to most β-lactam antibiotics. A novel and relatively common phenotype, termed NaHCO_3_ responsiveness, has been identified in which MRSA strains show increased susceptibility *in vitro* and *in vivo* to β-lactams in the presence of NaHCO_3_. A recently described S. aureus NaHCO_3_ transporter, MpsAB, is involved in intracellular NaHCO_3_ concentration for anaplerotic pathways. We investigated the role of MpsAB in mediating the NaHCO_3_ responsiveness phenotype in four prototype MRSA strains (two responsive and two nonresponsive). We demonstrated that MpsABC is an important contributor to the NaHCO_3_–β-lactam responsiveness phenotype. Our study adds to the growing body of well-defined characteristics of this novel phenotype, which could potentially translate to alternative targets for MRSA treatment using β-lactams.

## INTRODUCTION

Methicillin-resistant Staphylococcus aureus (MRSA) infections are a growing public health threat worldwide and the cause of a number of serious infections, including bacteremia, endocarditis, osteomyelitis, and invasive skin and soft tissue infections (1, 2). A major concern with MRSA therapies is the *in vitro* resistance of these organisms to most β-lactam agents (except ceftaroline and ceftobiprole) (3) as well as other antibiotics (e.g., aminoglycosides and quinolones).

One recent goal in clinical microbiology has been to improve standardized antimicrobial susceptibility testing (AST) for resistant bacteria, such as MRSA, by modifying *in vitro* growth conditions to better reflect the host physiologic microenvironment (4). In MRSA, such AST modifications have identified a novel phenotype in a relatively large proportion of clinical MRSA isolates, known as NaHCO_3_–β-lactam responsiveness. Such isolates exhibit enhanced susceptibility (i.e., ≥4-fold reductions in MICs) to early-generation β-lactams, such as cefazolin and oxacillin, when grown *in vitro* in the presence of NaHCO_3_ supplementation (4–7). According to the Clinical and Laboratory Standards Institute (CLSI) guidelines, MIC breakpoints for oxacillin are interpreted as susceptible (S) at ≤2 μg/mL and resistant (R) at ≥4 μg/mL for S. aureus (8).

Recent investigations from our laboratory have defined a number of key phenotypic and genotypic characteristics of MRSA strains that distinguish NaHCO_3_-responsive from NaHCO_3_-nonresponsive isolates when exposed to NaHCO_3_, including (i) reduced production of PBP2a (a major determinant of β-lactam resistance); (ii) reduced expression of *mecA* (a gene responsible for PBP2a production), *blaZ* (coregulator of PBP2a production), and *sarA* (required for maintenance of the MRSA phenotype); (iii) reduced expression of *pbp4*, *vraS*, and *prsA* (required for intramembrane maturation and final folding of PBP2a); (iv) a synergistic impact on killing when NaHCO_3_ is combined with host defense cationic peptides; (v) increased β-lactam binding to the MRSA surface and to membrane-localized PBP2a; (vi) the presence of specific genotypes within the ribosome binding site (RBS) and coding regions of *mecA* (e.g., 246G versus 246E); (vii) an association with the combination of *in vitro* susceptibility to amoxicillin-clavulanate, specific *mecA* susceptible genotypes (246G), and specific *spa* types (t002 or t008); (viii) reductions in wall teichoic acid (WTA) synthesis via posttranslational mechanisms (e.g., gene products involved in peptidoglycan synthesis and functionality); and (ix) enhanced clearance from simulated cardiac vegetations *ex vivo*, as well as from vegetations and other target tissues in experimental endocarditis *in vivo* (4, 6, 7, 9–14).

Importantly, a NaHCO_3_ transporter named MpsAB was recently identified in S. aureus, the first example of such transporters in the phylum *Firmicutes* and other nonautotrophic bacteria (15). It is encoded by the *mpsABC* operon, which was initially characterized for its functionality as a key membrane potential-generating system (16). Although the small *mpsC* gene is part of the operon, it apparently does not contribute to NaHCO_3_ transport. MpsAB has now been confirmed as the principal determinant of NaHCO_3_ transport in S. aureus (15). Of note, such NaHCO_3_ cotransporters are widespread in both autotrophic and nonautotrophic bacteria. As the only dissolved inorganic carbon supply system in S. aureus, MpsAB plays an important role in concentrating bicarbonate for key anaplerotic pathways (15, 17, 18). Deletion of *mpsAB* genes in S. aureus leads to severe growth delay under ambient-air conditions, which is reversible only by NaHCO_3_ or CO_2_ supplementation (15).

Many investigations have been done independently on NaHCO_3_ responsiveness (4–7) and on the NaHCO_3_ transport systems (15, 17–19). However, the interplay between these two factors remains unknown. We postulated that these phenotypic differences in NaHCO_3_ responsiveness among MRSA strains might be impacted, at least in part, by either structural or functional characteristics of the MpsAB transporter.

The aims of the current study were to compare the role of the NaHCO_3_ transporter MpsAB in mediating the NaHCO_3_–β-lactam responsiveness phenotype using four prototype MRSA strains (two NaHCO_3_-responsive and two NaHCO_3_-nonresponsive strains). We compared strains with these two distinct MRSA phenotypes for (i) the amino acid sequence of their MpsAB transporters; (ii) NaHCO_3_ uptake in ambient air versus in CO_2_, using radiolabeled NaH^14^CO_3_; (iii) the effect of *mpsABC* deletions on oxacillin MICs; and (iv) the effects of NaHCO_3_-oxacillin exposure on *mpsA* transcription and translation analyzed by quantitative reverse transcription-PCR (qRT-PCR) and flow cytometry.

## RESULTS

### NaH^14^CO_3_ uptake is significantly higher in NaHCO_3_-responsive strains vs NaHCO_3_-nonresponsive MRSA.

**FIG 1:**
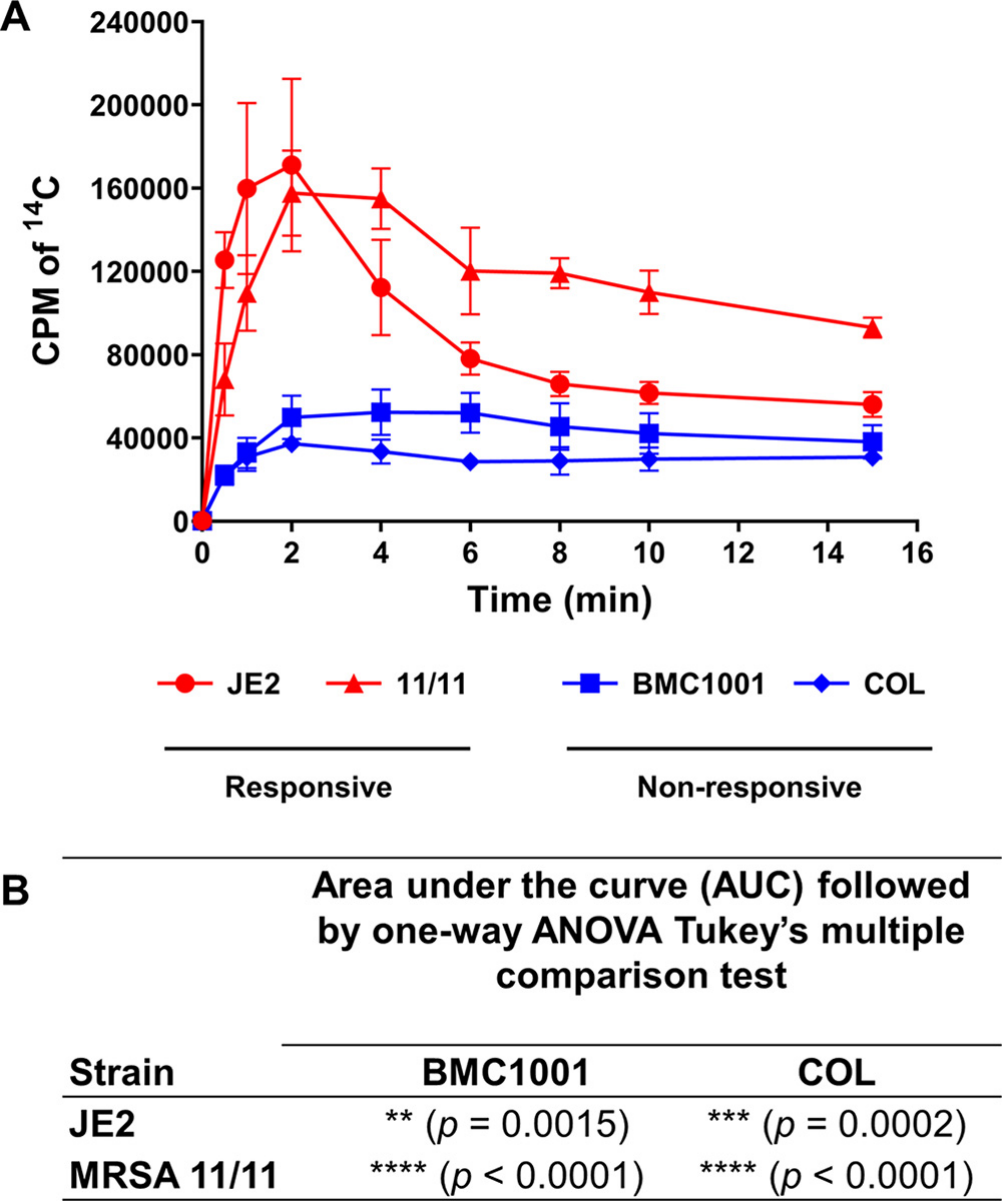
(A) Uptake of NaH^14^CO_3_ by bicarbonate-responsive strains (S. aureus JE2 and MRSA 11/11; red lines and symbols) and nonresponsive strains (S. aureus BMC1001 and COL; blue lines and symbols). Bacterial cultures grown in ambient air until mid-exponential phase were washed and adjusted to the same OD. Fluorocitrate was added to the cells and incubated for 30 min before the addition of NaH^14^CO_3_ (50 μCi). Aliquots of cell suspensions were collected at the indicated time points, and the H^14^CO_3_ uptake was determined by the ^14^C accumulation in cells, as measured by liquid scintillation counting. Each value is the mean and SD from three independent biological replicates. (B) The *P* values represent the significant differences in the AUC for the uptake of each strain, analyzed using one-way ANOVA followed by Tukey’s multiple-comparison test. The H^14^CO_3_ uptake in the responsive strains JE2 and MRSA 11/11 was significantly higher than that in the nonresponsive strains BMC1001 and COL.

Our previous studies demonstrated that a substantial proportion of clinical MRSA strains exhibit enhanced susceptibility *in vitro* to early-generation β-lactams (e.g., oxacillin and cefazolin) when grown in the presence of NaHCO_3_ (4–6). To evaluate if the known NaHCO_3_ transporter MpsAB plays a role in NaHCO_3_ responsiveness, we compared the NaHCO_3_ uptake activity in two NaHCO_3_-responsive strains (JE2 and MRSA 11/11) and two NaHCO_3_-nonresponsive strains (BMC1001 and COL) using radiolabeled NaH^14^CO_3_. Prior to the addition of NaH^14^CO_3_, fluorocitrate and glucose were added to the cell suspensions. Fluorocitrate, an aconitase inhibitor, was added to prevent the rapid expiration of CO_2_ by the decarboxylation reactions of the tricarboxylic acid (TCA) cycle (15). The NaH^14^CO_3_ uptake occurred almost immediately, peaking within 2 min, before decreasing to a steady-state level at the end of 15 min (Fig. 1A). The total NaH^14^CO_3_ uptake under ambient-air conditions for each strain was calculated from the area under the time-accumulation curve (AUC). These studies showed that the NaH^14^CO_3_ uptake was highest in the two NaHCO_3_-responsive strains, MRSA 11/11 and JE2, and lowest in the two NaHCO_3_-nonresponsive strains, BMC1001 and COL (Fig. 1A). Statistical analyses revealed that the responsive strains, JE2 and MRSA 11/11, had significantly higher H^14^CO_3_ uptake than the nonresponsive strains BMC1001 and COL (Fig. 1B).

### NaHCO_3_-responsive strains show lower NaH^14^CO_3_ uptake when grown in 5% CO_2_ than in ambient air.

**FIG 2:**
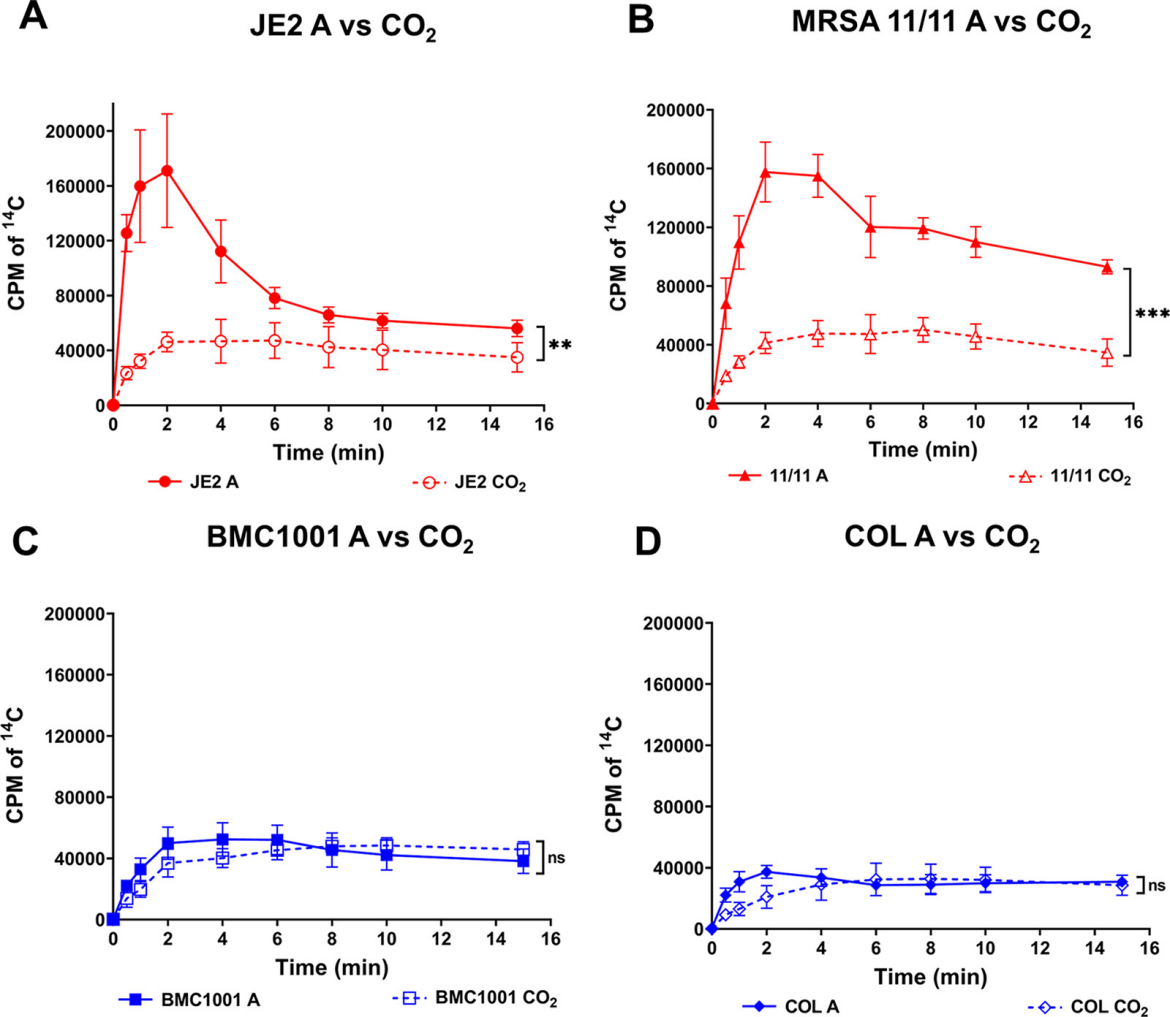
Uptake of NaH^14^CO_3_ by strain sets grown in ambient air (A; solid lines and symbols) and 5% CO_2_ conditions (CO_2_; dashed lines and symbols). The bicarbonate-responsive strains (A) JE2 and (B) MRSA 11/11 showed significantly higher H^14^CO_3_ uptake when grown in ambient air than in CO_2_. **, *P = *0.0092; ***, *P = *0.0003. The H^14^CO_3_ uptake for cells grown in ambient air and 5% CO_2_ was not significantly different in the nonresponsive strains (C) BMC1001 (ns, not significant [*P = *0.7847]) and (D) COL (*P = *0.6652). Each point represents the mean and SD from three independent biological replicates. Statistical significance between the uptake in ambient air and CO_2_ was calculated using Student’s *t* test from the AUC values for each strain set.

To assess the influence of CO_2_ on NaH^14^CO_3_ uptake, the above studies were carried out in parallel in the presence of 5% CO_2_. As shown in Fig. 2, both responsive strains showed significantly lower NaH^14^CO_3_ uptake when grown with CO_2_ (2.2 and 2.8 times lower for JE2 and MRSA 11/11, respectively) (Fig. 2A and B). In contrast, the two nonresponsive strains (BMC1001 and COL) showed no significant differences in the NaH^14^CO_3_ uptake when grown in ambient air versus CO_2_ (Fig. 2C and D). This suggests the possibility that the *mpsAB* NaHCO_3_ uptake system in responsive strains is partially repressed by an exogenous supply of CO_2_ (as it is not required in the latter scenario); on the other hand, this uptake system is more constitutive in nonresponsive strains and is not repressible by exogenous CO_2_.

### *mpsABC* sequences are highly similar in all four NaHCO_3_-responsive and NaHCO_3_-nonresponsive MRSA strains.

Since differences were seen in the NaHCO_3_ uptake under ambient-air and CO_2_ growth conditions, we examined whether the *mpsABC* operon exhibited substantial differences in the four study strains. Multiple-sequence alignment of the *mpsABC* locus of JE2, MRSA 11/11, BMC1001, and COL showed that there are only two mismatches out of 4,727 bp, resulting in 99.4% similarity among the four prototype strains. In both BMC1001 and COL, there is a nucleotide change from T to C in the coding region of *mpsB*, causing a change in the 264th amino acid position of MpsB from tyrosine to histidine. Additionally, COL has another nucleotide change, from C to A, resulting in the change in the 466th amino acid of MpsA from alanine to glutamic acid (see Fig. S1A in the supplemental material). The promoter sequences of *mpsA* were 100% identical among the four prototype strains. To verify whether the two single nucleotide polymorphisms (SNPs) found in the *mpsABC* locus of the nonresponsive strains are determinative of the latter phenotype, we introduced these SNPs into the responsive strain backgrounds by “swapping” the *mpsABC* region from COL into JE2 and MRSA 11/11 (Fig. S1B); this generated JE2 and MRSA 11/11 swap variants which harbor both SNPs. The oxacillin MICs were then checked using these swap variants to see whether the phenotype switched from oxacillin-NaHCO_3_ responsiveness to nonresponsiveness. The oxacillin MIC data showed that both the JE2 and MRSA 11/11 swap variants harboring the SNPs from nonresponsive strain COL remained responsive to NaHCO_3_ (Table S1).

### Growth studies of prototype strains.

In our previous studies, NaHCO_3_ supplementation had a minimal impact on the 24-h growth kinetics of our four prototype strains used in the current investigation. Thus, the 24-h growth yields were not significantly different for growth in cation-adjusted Mueller-Hinton broth (CA-MHB)–Tris medium with NaHCO_3_ versus growth in the same medium without NaHCO_3_ for any strain tested except COL, which grew somewhat more slowly than the other three strains (4). The long doubling time of COL was documented previously (20). Growth yields of these four prototype parental strains at 24 h in ambient air versus 5% CO_2_ did not show any major differences (Fig. S2).

### Deletion of *mpsABC* reversed the NaHCO_3_-responsive phenotype in responsive strains, determined by oxacillin MIC testing.

To determine whether a functional NaHCO_3_ transporter affected the NaHCO_3_-responsive phenotype, *mpsABC* deletion mutants and their respective plasmid complementation constructs were constructed in the four prototype MRSA strains; these strain sets were then assessed for oxacillin MICs in the presence versus the absence of NaHCO_3_ under either ambient-air or 5% CO_2_ growth conditions. CA-MHB–Tris alone was used as the control (4).

In ambient air, the two responsive parental strains showed the expected NaHCO_3_ responsiveness phenotype, with 64-fold reductions in oxacillin MICs in ambient air in the presence of NaHCO_3_ (Table 1). The ambient-air oxacillin MICs for the deletion mutants and complemented variants could not be obtained, because these constructs grew very poorly under such conditions.

**TABLE 1:** MICs of oxacillin in NaHCO_3_-responsive and -nonresponsive MRSA strains^*a*^

Strain	Oxacillin MIC (μg/mL)			
	Ambient air		5% CO_2_	
	CA-MHB–Tris	CA-MHB–Tris + 44 mM NaHCO_3_	CA-MHB–Tris	CA-MHB–Tris + 44 mM NaHCO_3_
Responsive				
JE2	64	1	8	1
JE2 Δ*mpsABC*	—	—	16	16
JE2 Δ*mpsABC* compl.	—	—	16	1
MRSA 11/11	32	0.5	2	1
MRSA 11/11 Δ*mpsABC*	—	—	4	8
MRSA 11/11 Δ*mpsABC* compl.	—	—	1	0.5
Nonresponsive				
BMC1001	256	512	64	128
BMC1001 Δ*mpsABC*	—	—	32	64
BMC1001 Δ*mpsABC* compl.	—	—	32	64
COL	256	512	64	128
COL Δ*mpsABC*	—	—	64	128
COL Δ*mpsABC* compl.	—	—	64	128

a CA-MHB–Tris, cation-adjusted Mueller-Hinton broth supplemented with 100 mM Tris maintained at pH ~7.2; —, the MIC was not obtained because the growth was severely affected in ambient air; compl., complemented. All MHB used was supplemented with 2% NaCl for testing with oxacillin. MICs were obtained from at least three independent biological replicates.

In the presence of 5% CO_2_, the baseline MICs of both responsive parental strains were lower, but each displayed a 2- to 8-fold reduction in oxacillin MICs in the presence of NaHCO_3_ supplementation. In contrast, in the *mpsABC* deletion mutants of these two responsive strains, the NaHCO_3_-responsive phenotype was not observed. NaHCO_3_ responsiveness was restored in both complementation constructs in the presence of NaHCO_3_ supplementation (Table 1).

For the nonresponsive strains, oxacillin MICs were unaffected when the two parental strains were compared with their respective deletion or complementation mutants, in the presence versus the absence of NaHCO_3_ supplementation, whether in ambient air or 5% CO_2_ (Table 1).

### Transcription and translation of *mpsA* are increased by NaHCO_3_ exposure in responsive versus nonresponsive strains.

**FIG 3:**
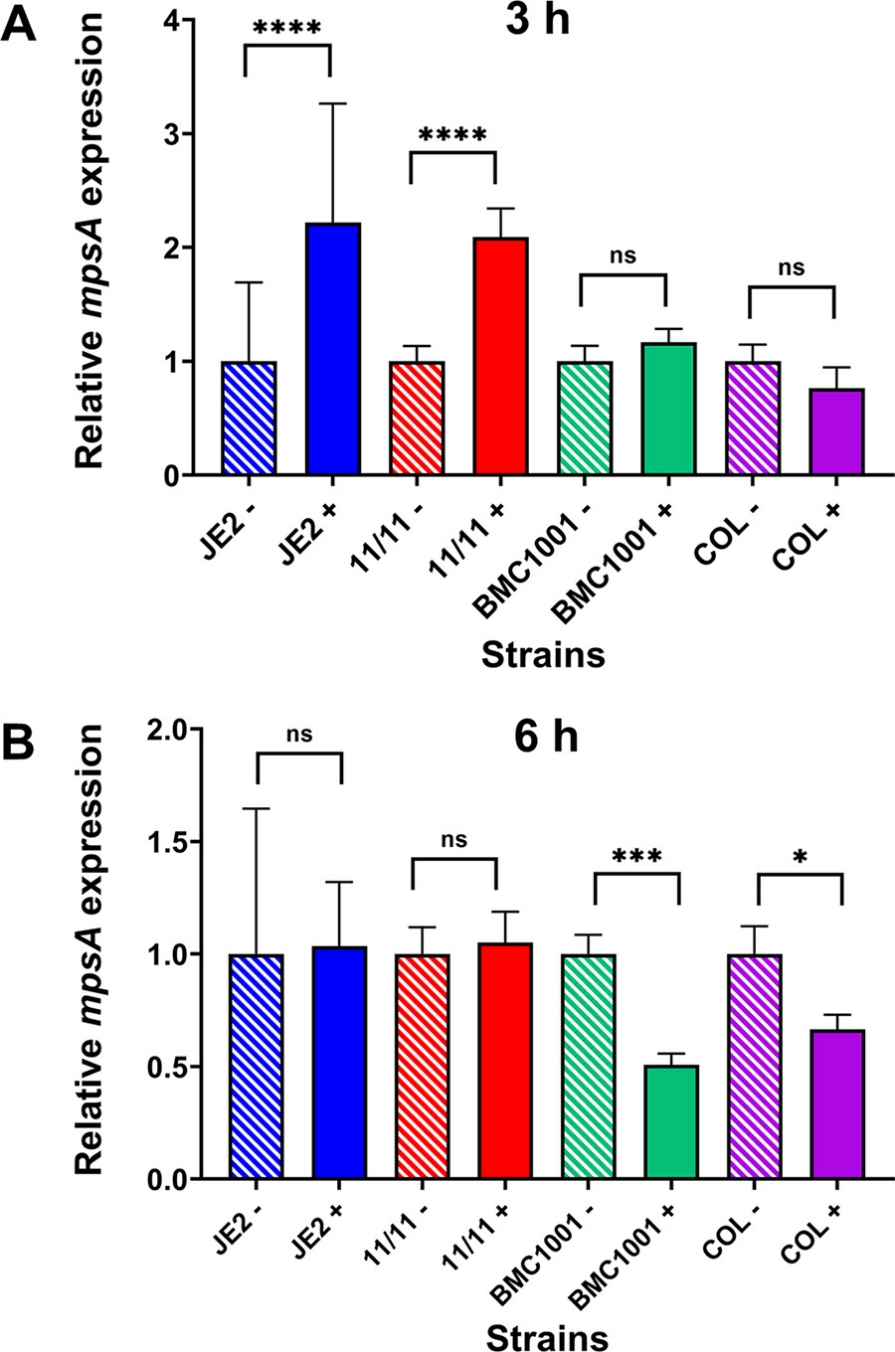
Expression of *mpsA* in NaHCO_3_-responsive (JE2 and MRSA 11/11) and NaHCO_3_-nonresponsive (BMC1001 and COL) strains. Gene expression data were obtained by qRT-PCR of RNA from (A) mid-exponential-phase (3 h) and (B) early-stationary-phase (6 h) strains grown in CA-MHB–Tris with (+) or without (−) NaHCO_3_ and 1/2 MIC of oxacillin. NaCl (2%) was included in growth media in which oxacillin was also included. For each strain, *mpsA* expression was normalized to the value obtained in CA-MHB–Tris (−), with this value set to 1.0. Data are means and SD from three independent biological replicates. Statistical comparisons were determined by Student’s *t* test. ns, not significant; *, *P* < 0.05; ***, *P* < 0.001; ****, *P* < 0.0001.

During mid-exponential phase, by qRT-PCR, *mpsA* expression, as the first gene of the *mpsABC* operon, was significantly upregulated (~2-fold) in both responsive strains (JE2 and MRSA 11/11) by NaHCO_3_ supplementation in the presence of oxacillin. In contrast, NaHCO_3_ had no impact on *mpsA* expression in the two nonresponsive strains at this growth phase (Fig. 3A). In early stationary phase, in the two responsive strains, NaHCO_3_ supplementation had no impact on *mpsA* expression, while *mpsA* expression was reduced 2-fold in nonresponsive strains (Fig. 3B). As a control, the addition of oxacillin alone (i.e., in the absence of NaHCO_3_) did not have any effect on the expression of *mpsA* at either growth phase (Fig. S3).

For confirmation of the impact of NaHCO_3_-oxacillin exposure on *mpsA* transcription, as well as on translation, strategic *mpsA*-green fluorescent protein (GFP) fusion constructs were assessed by flow cytometry. Transcriptional (txn) constructs carry the promoter region of the *mpsA* with the heterologous *sarA* RBS fused to the *gfp* gene; translational (tln) constructs harbor the promoter region of the *mpsA* with its intrinsic *mpsA* RBS fused to the *gfp* gene (Fig. S4). As noted in Materials and Methods, the two flow-cytometric readouts which were assessed and quantitatively compared were (i) percent GFP fluorescence, indicating the proportion of the 10,000-cell population that expressed GFP above the control baselines for the strain set, and (ii) the mean fluorescence intensity (MFI), measuring the mean per-cell GFP expression.

**FIG 4:**
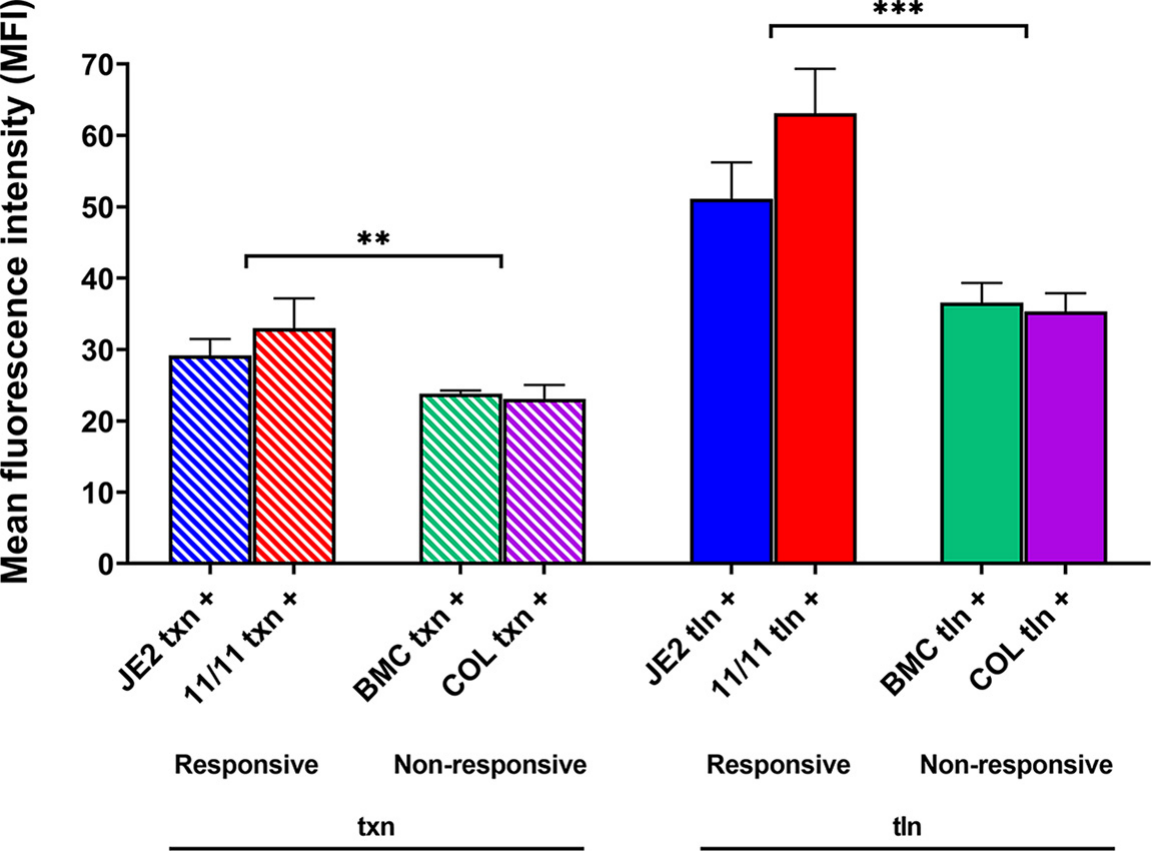
Translational efficiency of *mpsA* promoter sequences for all the prototype strains in the presence of NaHCO_3_ and oxacillin. Translational efficiency was assessed by flow cytometry using strains harboring the promoter-GFP fusions (Fig. S4) for JE2, MRSA 11/11, BMC1001, and COL promoter regions. Transcriptional (txn) constructs harbor the promoter region of *mpsA* with the *sarA* RBS fused to *gfp* (left), while translational (tln) constructs harbor the promoter region of *mpsA* with the *mpsA* RBS fused to *gfp* (right). Cells were grown in CA-MHB–Tris, 1/2 MIC of oxacillin (16 μg/mL for JE2 and MRSA 11/11, 64 μg/mL for BMC1001, and 128 μg/mL for COL), and 2% NaCl for 3 h before being assessed for MFI via flow cytometry. NaHCO_3_ (44 mM) was added to all the media (+). Statistical significance of the MFI between the responsive and nonresponsive strains was determined by Student’s *t* test. **, *P = *0.0044; ***, *P = *0.0003. Data are the results of four independent biological replicates for each strain/condition.

**FIG 5:**
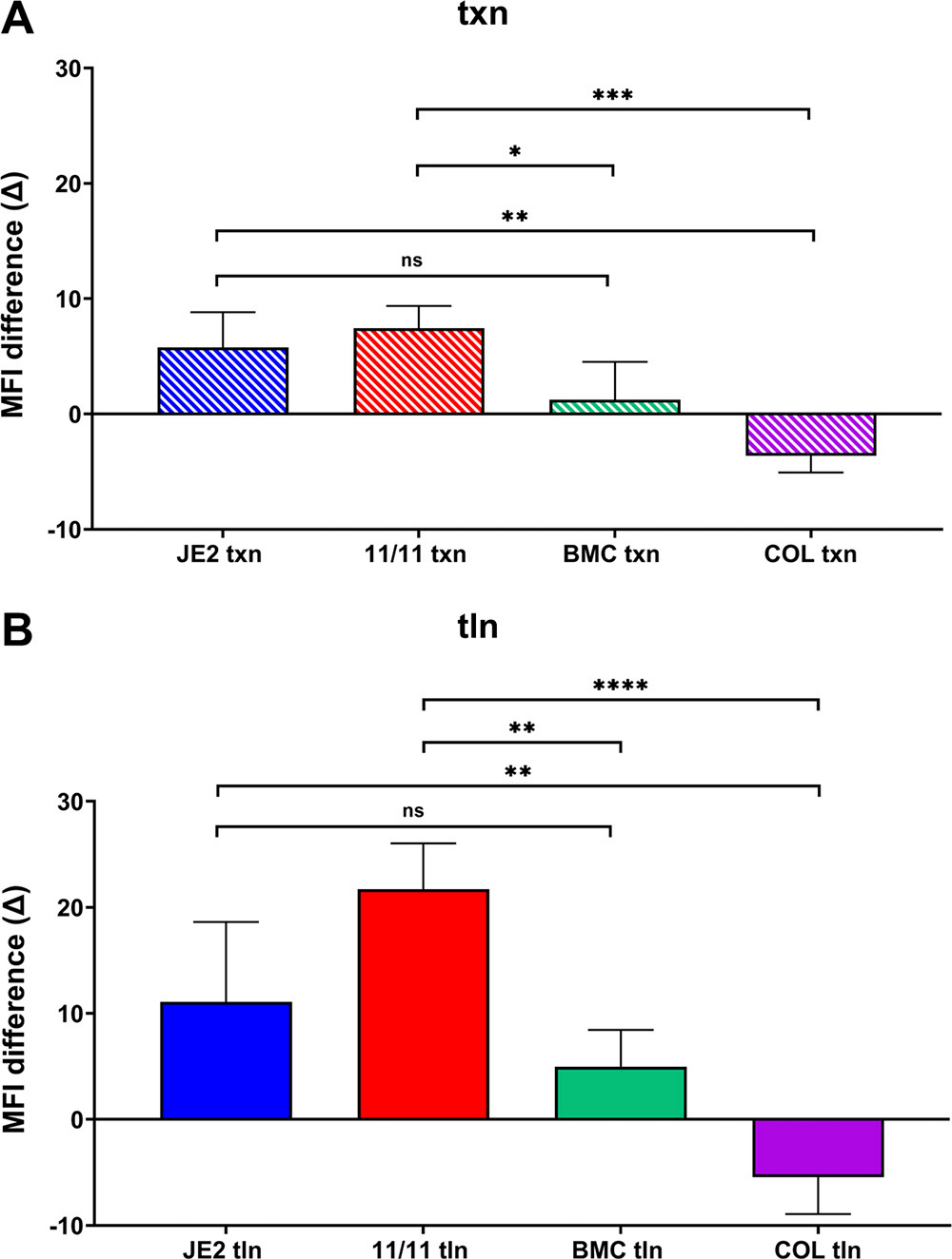
Differences in MFI of strains JE2, MRSA 11/11, BMC1001, and COL grown with and without NaHCO_3_ in the presence of 1/2 MIC of oxacillin for (A) transcriptional (txn) and (B) translational (tln) constructs. The constructs and conditions used are indicated in the legend to Fig. 4. Each bar shows the difference (increase/decrease) between the MFIs of the strain grown with and without NaHCO_3_ (baseline levels), and the statistical significance between them was calculated using one-way ANOVA followed by Tukey’s multiple-comparison test. ns, not significant (*P > *0.05); *, *P < *0.05; **, *P < *0.01; ***, *P < *0.001; ****, *P < *0.0001. Data are the results from four independent biological replicates for each strain/condition.

Consistent with the qRT-PCR data shown in Fig. 3, MFI data also demonstrated that the two responsive strains had significantly higher GFP expression at both txn and tln levels after exposure to NaHCO_3_-oxacillin than the two nonresponsive strains (Fig. 4). This difference was even more impressive when the changes in MFI metrics (ΔMFI) in the responsive and nonresponsive strains in the absence (baseline with oxacillin alone) versus the presence of NaHCO_3_-oxacillin exposure were compared (Fig. 5A).

As expected, the tln metrics (utilizing MFI readouts) closely paralleled those observed in the txn assays mentioned above. Thus, in both responsive strains, the MFI txn metrics were significantly higher than those in the nonresponsive strains (Fig. 4). These tln differences were further magnified when baseline expression in the absence of NaHCO_3_ was compared with expression in the presence of NaHCO_3_-oxacillin exposure for the two phenotype groups (Fig. 5B).

In parallel, we assessed the percent GFP expression for both the txn and tln constructs, in the presence and the absence of NaHCO_3_-oxacillin exposure. As noted above, this metric indicates the proportion of the overall 10,000 cell population tested by flow cytometry expressing GFP above the control, background gated level. In contrast to the MFI data detailed above, there were essentially no differences in the percent GFP data sets comparing the responsive versus nonresponsive strain groups (Fig. S5 and S6). This indicated that the same proportion of the cell populations for each phenotype group transcribed and translated the *mpsA* message; the differences between the phenotype groups appear to depend upon the extent to which txn and tln proceed (Fig. S6). As a control, the addition of oxacillin alone (i.e., in the absence of NaHCO_3_) did not have any influence on the txn and tln efficiency of each corresponding strains (Fig. S7 and S8).

## DISCUSSION

The overall goal of the current study was to investigate the effect of the *mpsABC* NaHCO_3_ uptake system on the NaHCO_3_-responsive and NaHCO_3_-nonresponsive MRSA phenotypes in prototype strains. A number of interesting outcomes were observed when MRSA strains which are NaHCO_3_ responsive were compared with those that are not, as defined *in vitro*. First, among our prototype MRSA strain set, the *mpsABC* coding sequences were essentially identical, with only two amino acid substitutions. The promoter sequences of *mpsA* also shared 100% identity among the prototype strain set. The oxacillin MIC data for the JE2 and MRSA 11/11 swap variants confirmed that the two SNPs contained within the *mpsABC* coding region do not dictate NaHCO_3_ responsiveness phenotype. The *mpsAB* SNPs found in nonresponsive strains seem to yield this phenotype only in selected genetic backgrounds, implying that regulatory factors beyond the *mpsABC* operon are in play. In addition to the above-mentioned reasoning, the high degree of identity in the *mpsABC* sequence and its promoter in all four prototype strains suggests that the phenotypic differences in NaHCO_3_-responsive versus NaHCO_3_-nonresponsive MRSA strains were likely due to genes or pathways outside *mpsABC* that impacted its functionality and/or posttranslational modifications of the MpsABC transporter.

Second, deletions of *mpsABC* caused loss of the NaHCO_3_-responsive, oxacillin-susceptible phenotype in both intrinsically responsive MRSA strains (a defect which was plasmid complementable) but had no impact in the intrinsically nonresponsive strains. Of importance, the deletion mutants of both responsive and nonresponsive strains could not grow in ambient air; in contrast, all deletion mutants grew well in the presence of 5% CO_2_. The latter phenomenon is expected, since CO_2_ can diffuse freely into the MRSA cell, although the rate and extent of this diffusibility can be substantially impacted by the degree of biofilm formation in a given strain (17). One notable observation was that CO_2_ supplementation was able to suppress NaHCO_3_ uptake in both responsive strains but not in either nonresponsive strain. There are two possibilities here. (i) It may be that in NaHCO_3_-responsive strains, a feedback inhibition system recognizes the availability of exogenous CO_2_ and is able to repress the *mpsABC* NaHCO_3_ uptake system; in contrast, this uptake system is relatively constitutive in nonresponsive strains but at a much lower setpoint. (ii) The intracellular CO_2_ fixation systems may be quantitatively or functionally different in NaHCO_3_-responsive versus -nonresponsive strains.

This concept of fixation of CO_2_ is that this molecule, which is small and uncharged, moves freely into and out of the cell, based only on concentration differences. On the other hand, HCO_3_^−^ is a charged molecule which remains within the organism once taken up. While CO_2_ can spontaneously be converted to HCO_3_^−^ in an aqueous environment, the reverse reaction occurs as well. In many organisms, an enzyme with an extremely high turnover number can rapidly convert CO_2_ to HCO_3_^−^ intracellularly, thereby capturing it inside the cell. Carbonic anhydrase is not present in S. aureus; thus, for S. aureus to retain CO_2_ (i.e., fix it), the spontaneously produced HCO_3_^−^ must be rapidly integrated into an intracellular molecule. As examples, two enzymes, PcyA and PurK, fix HCO_3_^−^ intracellularly by carboxylating pyruvate and 5-amino-1-(5-phospho-d-ribosyl)imidazole, respectively. This prevents CO_2_ from diffusing out of the cell, thereby enhancing growth. Pyruvate carboxylase adds HCO_3_^−^ to pyruvate to form oxaloacetate, which then feeds the TCA cycle; importantly, the TCA cycle is linked to the cytochrome system, which can also help produce a membrane potential. Thus, even when *mpsABC* is deleted, intracellular CO_2_ can help generate a membrane potential, albeit at a much lower level (15).

Third, NaHCO_3_-responsive MRSA took up NaHCO_3_ more quickly and to a greater extent than NaHCO_3_-nonresponsive strains (over a 15-min period); these early uptake differences roughly correlate with higher early growth rates in NaHCO_3_-responsive than NaHCO_3_-nonresponsive MRSA strains. The β-lactam antibiotics are more active during accelerated growth, which correlates with upregulated ATP production to compensate for the large amounts being consumed during cell wall biosynthesis.

Fourth, our data show differences in NaHCO_3_ uptake profiles of NaHCO_3_-responsive and -nonresponsive strains; responsive strains exhibited enhanced NaHCO_3_ uptake under ambient-air conditions. In this study, we found that CO_2_ blocks the uptake of HCO_3_ in responsive versus nonresponsive strains. There are many metabolic pathways in S. aureus that require CO_2_, so defining which specific reaction is pivotal is not possible at this time. However, from our data, we can make some predictions about glutamine metabolism impacting maintenance of the methicillin resistance (MRSA) phenotype (i.e., HCO_3_ nonresponsiveness). Several lines of evidence suggest that responsive strains are not able to generate sufficient endogenous CO_2_ under ambient-air conditions (versus nonresponsive strains). (i) Endogenous production of CO_2_ is dependent upon intracellular glutamate levels and, to some extent, aspartate levels (21). Other amino acids and carbohydrates do not contribute to endogenous CO_2_ production. This paradigm was studied by labeling staphylococcal cells with ^14^C. Moreover, the addition of glutamate or glucose to the growth medium does not impact the release of ^14^CO_2_. (ii) In S. aureus, glutamate is shuttled to glutamine by glutamine synthase, GlnA, and then onto carbamoyl phosphate, which is metabolized by carbamate kinase (ArcC). This process ultimately produces HCO_3_, ATP, and NH_3_, which are the principal metabolic products reported by Ramsey (21). (iii) Others have also noted a connection between glutamine and expression/maintenance of the MRSA phenotype (22). Of course, the regulator of the glutamine synthase operon, GlnR (also called FemC), is an accessory factor, which itself is required for expression of the MRSA phenotype (23). (iv) Finally, there are differences between the *arc* genes (important determinants within the glutamine biosynthetic pathway) of the USA300 (MRSA 11/11) and USA400 (MW2) clonal lineages (designated *arcC*) versus strains of the USA500 lineage (BMC1001) (designated *arc2*) (24, 25). Of note, the USA300 and USA400 lineages represent the two HCO_3_-responsive strains in our study, while the USA500 lineage represents one of our HCO_3_-nonresponsive strains. Thus, there are several lines of evidence to link glutamate metabolism, CO_2_, and the maintenance of expression of MRSA (i.e., a HCO_3_-nonresponsive state). We recognize (i) the complexity of the endogenous CO_2_-generating systems as outlined above and (ii) the somewhat speculative nature of our hypothesis (i.e., that HCO_3_-responsive strains need to take up more exogenous HCO_3_). We plan to investigate these CO_2_-generating systems further in future experiments.

Fifth, two additional factors that undoubtedly impact the differences in NaHCO_3_ uptake profiles seen in NaHCO_3_-responsive versus NaHCO_3_-nonresponsive strains are the expression (transcription) and translation metrics of the *mpsA* gene itself in the presence of exogenous NaHCO_3_. In the current study, when baseline expression was compared with NaHCO_3_-stimulated expression, the transcription of this gene during log phase was substantially increased by exogenous NaHCO_3_ exposure in NaHCO_3_-responsive strains while remaining at near-baseline levels in NaHCO_3_-nonresponsive strains. As anticipated, at stationary phase of growth, when *mpsA* expression is minimal, exogenous NaHCO_3_ exposure exerted no enhancement of *mpsA* expression. Similar to the transcription outcomes above, the profiles of exogenous NaHCO_3_-stimulated translation of the *mpsA* message were significantly higher in NaHCO_3_-responsive than NaHCO_3_-nonresponsive strains.

Last, the transcriptional data above, showing increased expression of *mpsA* in NaHCO_3_-responsive versus NaHCO_3_-nonresponsive strains in the presence of exogenous NaHCO_3_, were confirmed by both qRT-PCR and flow cytometry. This technique provided an additional key piece of information to this metric. These flow cytometry data demonstrated that the overall population of cells expressing this gene was not significantly different in the two phenotype groups; however, the quantitative per-cell expression (MFI) of this gene was significantly amplified by exogenous NaHCO_3_ exposure in NaHCO_3_-responsive versus NaHCO_3_-nonresponsive MRSA cells.

These investigations have several limitations. Our study strain set, although consisting of well-characterized and previously published strains, was relatively small. Future studies will be required to assess the *mpsABC* uptake system in a larger MRSA strain cohort. Also, the identification of genes/pathways which can significantly impact the *mpsABC* system, especially to explain the differential phenotypic and genotypic outcomes in NaHCO_3_-responsive versus -nonresponsive strains, will be required. Transcriptome sequencing (RNA-seq) analyses comparing parental JE2 versus JE2 *mpsABC* deletion constructs are in progress to help answer this question. Preliminary expression profiles have revealed the upregulation of genes responsible for cell wall hydrolase/modification, transporters, and phage-related genes in the JE2 *mpsABC* mutant, with the most highly upregulated gene being *sceD*, a transglycosylase gene (unpublished data). SceD is a divisome-specific autolysin postulated to participate in peptidoglycan turnover, cell wall hydrolysis, and cell division; its inactivation results in impaired cell separation and clumping of bacterial cultures (26). This result is consistent with our previous and independently derived RNA-seq data, where it was demonstrated that NaHCO_3_ strongly represses *sceD* expression (11). Thus, high levels of intracellular NaHCO_3_ appear to repress *sceD* expression, whereas low levels of intracellular NaHCO_3_ (such as those seen in *mpsABC* mutants) result in upregulation of *sceD*. Finally, RNA-seq analyses in the JE2 *mpsABC* mutant showed a downregulation of genes related to cell wall-bound and secreted enzymes and protein and ion transporters, with the most downregulated genes encoding toxins such as phenol-soluble modulins. Given that many of these differentially expressed genes are linked to cell wall homeostasis, it is tempting to speculate that the absence of the NaHCO_3_ transporter MpsAB exerts most of its effects on the cell wall and its adaptations in distinct microenvironments.

Clearly, additional investigations will be required to assess the impacts of endogenous CO_2_ generation systems, inhibitors of individual components of the proton motive force (ΔΨ and ΔpH), as well as the proton transport systems (e.g., F_o_F_1_ ATPase). While MpsAB can establish a ΔΨ, growing S. aureus in the presence of 5% CO_2_ allows the organism to maintain its normal ΔΨ (15), probably because the presence of CO_2_ enhances pyruvate carboxylase activity, thereby increasing TCA activity, leading to F_o_F_1_ ATPase upregulation.

Importantly, the translatability of this uptake system to β-lactam hypersusceptibility *in vivo* is currently being examined in the experimental endocarditis model, using strategic deletion and complementation *mpsABC* variants of our prototype NaHCO_3_-responsive and -nonresponsive strains treated with oxacillin.

## MATERIALS AND METHODS

### Bacterial strains and growth conditions.

All the strains used in this study are listed in Table S2. The four prototype strains which form the basis for this investigation (JE2, MRSA 11/11, BMC1001, and COL) have been well characterized by our laboratory and others (27–30).

For cloning procedures, all staphylococcal and Escherichia coli strains were grown with aeration in basic medium (BM) (1% soy peptone, 0.5% yeast extract, 0.5% NaCl, 0.1% glucose, and 0.1% K_2_HPO_4_ [pH 7.2]), unless specified otherwise. All cultures were grown in baffled shake flasks (flask-to-medium ratio, 1:10). When applicable, the medium was supplemented with 10 μg/mL chloramphenicol for selected staphylococcal strains and 100 μg/mL ampicillin for E. coli strains.

### Bicarbonate uptake analysis.

Bicarbonate uptake analysis was performed as previously described (15). Briefly, overnight cultures cultivated in tryptic soy broth (TSB) in ambient air and 5% CO_2_ were inoculated to an optical density at 578 nm (OD_578_) of 0.1 and grown until mid-exponential phase for 3 h under their respective conditions. Cells were then washed with 10 mM Tris buffer (pH 7) before resuspension in the same buffer and adjusted to an OD_578_ of 1 in a final volume of 10 mL. Fluorocitrate (1 mM) and glucose (5 mM) were added to the cell suspensions prior to incubation for 30 min at room temperature with magnetic stirring. Next, 50 μCi of NaH^14^CO_3_ (specific activity, 40 to 60 mCi/mmol; PerkinElmer) was added. One-milliliter aliquots of each sample were collected before (time zero) and after the addition of NaH^14^CO_3_ and at 0.5, 1, 2, 4, 6, 8, 10, and 15 min. After the collection of each sample, cells were immediately filtered by vacuum filtration onto membrane filters (Pall GN-6 Metricel membrane disc filter; 0.45-μm pore size, 25-mm diameter) and washed with 10 mL of Tris NaCl buffer (10 mM Tris with 100 mM NaCl). Membrane filters were subsequently placed in a vial containing 10 mL of liquid scintillation cocktail (Ultima Gold; PerkinElmer). Radioactivity retained on the membrane filters was measured in a PerkinElmer liquid scintillation analyzer (Tri-Carb 2800TR). The H^14^CO_3_ uptake was determined by the accumulation of ^14^C in the cells and recorded as counts per minute.

### Multiple-sequence alignment.

*mpsABC* sequences for JE2 are found at locus tags B7H15_02370, B7H15_02375, and B7H15_02380 of the complete genome of S. aureus JE2 retrieved from the NCBI database (31) under the accession number CP020619 (https://www.ncbi.nlm.nih.gov/nuccore/CP020619.1/). For COL, the *mpsABC* sequences are found at locus tags SACOL0494, SACOL0495, and SACOL0496 of the complete genome of an early methicillin-resistant isolate, S. aureus COL, which was isolated in the 1960s (30) and sequenced in 2005 (32) under the accession number CP000046, also from the NCBI database. MRSA 11/11 (28) and BMC1001 (29) were sequenced by our group with the BioSample IDs SAMN17703515 and SAMN17703518, respectively. Both can be found in the NCBI database with the accession number PRJNA697971 (https://www.ncbi.nlm.nih.gov/bioproject/697971). *mpsABC* and its promoter sequences from all the four prototype strains were aligned using Clustal Omega (33).

### Construction of *mpsABC* deletion mutants and respective complementation plasmid construct variants in the prototype MRSA strains.

The oligonucleotides used in this can be found in Table S3. The *mpsABC* deletion mutants in the background of S. aureus MRSA 11/11, BMC1001, and COL were constructed as markerless deletions using allelic replacements as previously described (17). The upstream and downstream flanking regions of *mpsABC* are approximately 2 kb each, and the sequences are identical for all three strains. Briefly, the up- and downstream regions were amplified from the chromosomal DNA of MRSA 11/11. The amplified fragments were assembled using SmaI-linearized plasmid pBASE6 (34) via Gibson assembly (35) with Hi-Fi DNA assembly master mix (New England Biolabs). The resulting plasmid was transformed into chemically competent E. coli DC10B (36). The authenticated constructs were first introduced into S. aureus RN4220 via electroporation and then into MRSA 11/11, BMC1001, and COL. Mutagenesis was performed as previously described (37). Deletion of *mpsABC* was confirmed by chromosomal PCR and DNA sequencing of the PCR products.

Complementation of the *mpsABC* deletion mutants in all strains was performed using the plasmid pRB473 harboring *mpsABC* along with its native promoter from a previous study (16). The recombinant plasmid was transformed into competent Δ*mpsABC* mutants of MRSA 11/11, BMC1001, and COL via electroporation and confirmed with PCR.

### Construction of GFP reporters for *mpsA* transcription and translation.

To quantify transcriptional and translational activity of the *mpsA* genes under the control of a reporter gene, *gfp_uvr_*, the upstream promoter region was cloned with or without the RBS of *mpsA* before the translational start codon (ATG) of the *gfp* reporter gene (Fig. S4) in the shuttle plasmid pALC1484 (38). First, the pALC1484 vector was modified by removing the RBS along with the spacing region between the RBS and start codon (ATG) of the *gfp* gene and replaced with the RBS and spacing region of the *mpsA* gene using pairwise primers with flanking EcoRI and XbaI sites, and template DNA as pALC1484 by PCR. A 394-bp promoter fragment with flanking EcoRI and XbaI sites of the *mpsA* gene without the RBS was amplified by PCR and cloned into the modified pALC1484 and pALC1484 plasmids in E. coli IM08B (39). Final constructs were verified by enzymatic digestion and DNA sequencing, mobilized into strains JE2, MBC1001, MRSA 11/11, and COL by electroporation, and selected on tryptic soy agar (TSA) with chloramphenicol (10 μg/mL).

### Construction of the *mpsAB* region SNP swap variant.

To construct strains with interchange (swap) of the *mpsAB* region, a 5.5-kb DNA fragment was amplified that contained the intact *mpsAB* genes from COL by PCR using primers with flanking BamHI sites at both ends (Table S3). The DNA fragment was cloned into a temperature-sensitive shuttle vector, pMAD-X (13), and then selected in E. coli IM08B (39) for the correct construct. After verification by restriction digestion and DNA sequencing, the interchanged construct was introduced into JE2 and MRSA 11/11 by electroporation and selected on chloramphenicol (10 μg/mL)- and X-Gal (5-bromo-4-chloro-3-indolyl-β-d-galactopyranoside)-containing plates for blue colonies at 30°C. Plasmid DNA was isolated and digested with BamHI for the verification of the presence of DNA fragment in the respective construct in the strains. The construction of chromosomal mutations in the respective strain by recombination or two-point crossover was performed by a routine procedure as described previously (40). Briefly, two-point crossover of the *mpsAB* region was performed by temperature shift by growing the strains at 43°C with chloramphenicol, followed by subculturing at 30°C without any antibiotic. Cells were plated with and without chloramphenicol in the presence of X-Gal (40 μg/mL) for selection and incubated at 37°C. White/nonblue colonies were cross-streaked to select chloramphenicol-susceptible colonies for the potential two-point crossover clones or mutants. The mutants were verified by chromosomal PCR and DNA sequencing of the PCR product.

### MIC determinations.

The MICs of oxacillin were determined by broth microdilution according to the CLSI guidelines as previously described (4, 41, 42). Briefly, cells were grown overnight in the indicated medium (CA-MHB, CA-MHB–Tris, or CA-MHB–Tris plus 44 mM NaHCO_3_) and then diluted into the same medium containing 2-fold serial dilutions of oxacillin with 2% NaCl. The final concentration of the cells used were 5 × 10^5^ CFU/mL. Microtiter plates were incubated overnight at 37°C in ambient air or 5% CO_2_. The MIC was read as the first well in which visual turbidity was reduced compared to the no-drug control well. All MICs are the modes from at least six independent determinations.

### Assessment of *mpsA* promoter-GFP fusions by flow cytometry.

For flow cytometry, strains containing the plasmid pALC1484 with various promoter-GFP constructs (Table S2) were grown overnight in the indicated medium (CA-MHB–Tris with or without 44 mM NaHCO_3_) at 37°C with aeration and then diluted 1:10 into the same medium with or without 1/2 MIC of oxacillin (16 μg/mL for JE2 and 11/11, 64 μg/mL for BMC1001, and 128 μg/mL for COL) and 2% NaCl. Cells were incubated at 37°C with aeration and grown for 3 h to mid-exponential phase before being diluted 1:10 into phosphate-buffered saline (PBS), and 10,000 cells were analyzed for GFP production by flow cytometry with FACSCalibur (Becton Dickinson). The MFI and percentage of cells expressing GFP in each sample were determined with FlowJo software (version 10.8) using data obtained from the FL1-H channel and expressed as relative fluorescent units per cell population or percentage of cells expressing GFP per cell population, respectively. All samples were performed in four independent biological replicates.

### RNA isolation and qRT-PCR analysis of *mpsA* expression.

To quantify the combined effect of NaHCO_3_ exposure and oxacillin stimulation on the expression of *mpsA*, overnight cultures of the strains were grown in CA-MHB Tris with or without 44 mM NaHCO_3_ and 1/2 MIC of oxacillin, inoculated to an OD_578_ of 0.1, and grown for 3 h and 6 h, respectively. As a control to check if oxacillin alone affects the expression of *mpsA*, cultures were also grown in CA-MHB–Tris with or without 1/2 MIC of oxacillin for 3 h (Fig. S3). NaCl (2%) was included in growth media in which oxacillin was also included. The same amount of cells at a given OD_578_ was collected for all the strains and conditions and RNA was extracted from the cell pellets using FastPrep (FP120; Thermo Savant) with lysing matrix B tubes (MP Biomedicals). Total RNA was isolated by column purification (Qiagen), followed by DNase treatment (Turbo DNA-free; Invitrogen, Thermo Fisher Scientific) and then reverse transcribed to generate a cDNA library (Superscript IV; Invitrogen, Thermo Fisher Scientific). qRT-PCR was performed using a StepOne real-time PCR system (Applied Biosystems) with the primers listed in Table S3. *gyrB* was used as a housekeeping gene to normalize transcript quantifications. Relative quantification was carried out using the cycle threshold (ΔΔ*C_T_*) method. The data are presented as the fold change in *mpsA* expression in the presence of oxacillin and NaHCO_3_ compared to CA-MHB–Tris for each strain, with the latter being normalized to 1.0. All qRT-PCR gene expression data were determined from at least two independent biological replicates for each condition and tested in duplicate.

### Statistical analyses.

All data are presented as the sample means and standard deviations (SD), unless otherwise indicated. The bicarbonate uptake data were also quantified as the AUC for comparison among the strains under different conditions. All statistical analyses were carried out using Student’s *t* test for comparison between two groups, while one-way analysis of variance (ANOVA) followed by Tukey’s multiple-comparison test to compare the means among three or more groups. *P* values of <0.05 were considered statistically significant. All statistical analyses were performed using Microsoft Excel or GraphPad Prism 9 software.

### Data availability.

The main data supporting the findings of this work are available within the article and its supplemental material or from the corresponding author upon request.
