# Supplementary material for: Role of the NaHCO3 Transporter MpsABC in the NaHCO3–β-Lactam-Responsive Phenotype in Methicillin-Resistant Staphylococcus aureus
Source: Microbiol Spectr. 2023 Apr 27;11(3):e00141-23. doi: 10.1128/spectrum.00141-23 (PMC10269494; doi:10.1128/spectrum.00141-23)
Supplement: Supplemental file 1 — Supplemental material. Download spectrum.00141-23-s0001.pdf, PDF file, 0.9 MB [file spectrum.00141-23-s0001.pdf]

SUPPLEMENTARY MATERIALS

Role of the MpsABC $NaHCO_3$ transporter in the $NaHCO_3$-β-lactam-responsive phenotype in methicillin-resistant *Staphylococcus aureus* (MRSA)

Sook-Ha Fan,[a,#] Richard A. Proctor,[b,c] Selvi C. Ersoy,[a] Adhar C. Manna,[d] Ambrose L. Cheung,[d] Friedrich Goetz,[e] Henry F. Chambers,[f] and Arnold S. Bayer[a,g,#]

SUPPLEMENTARY FIGURES

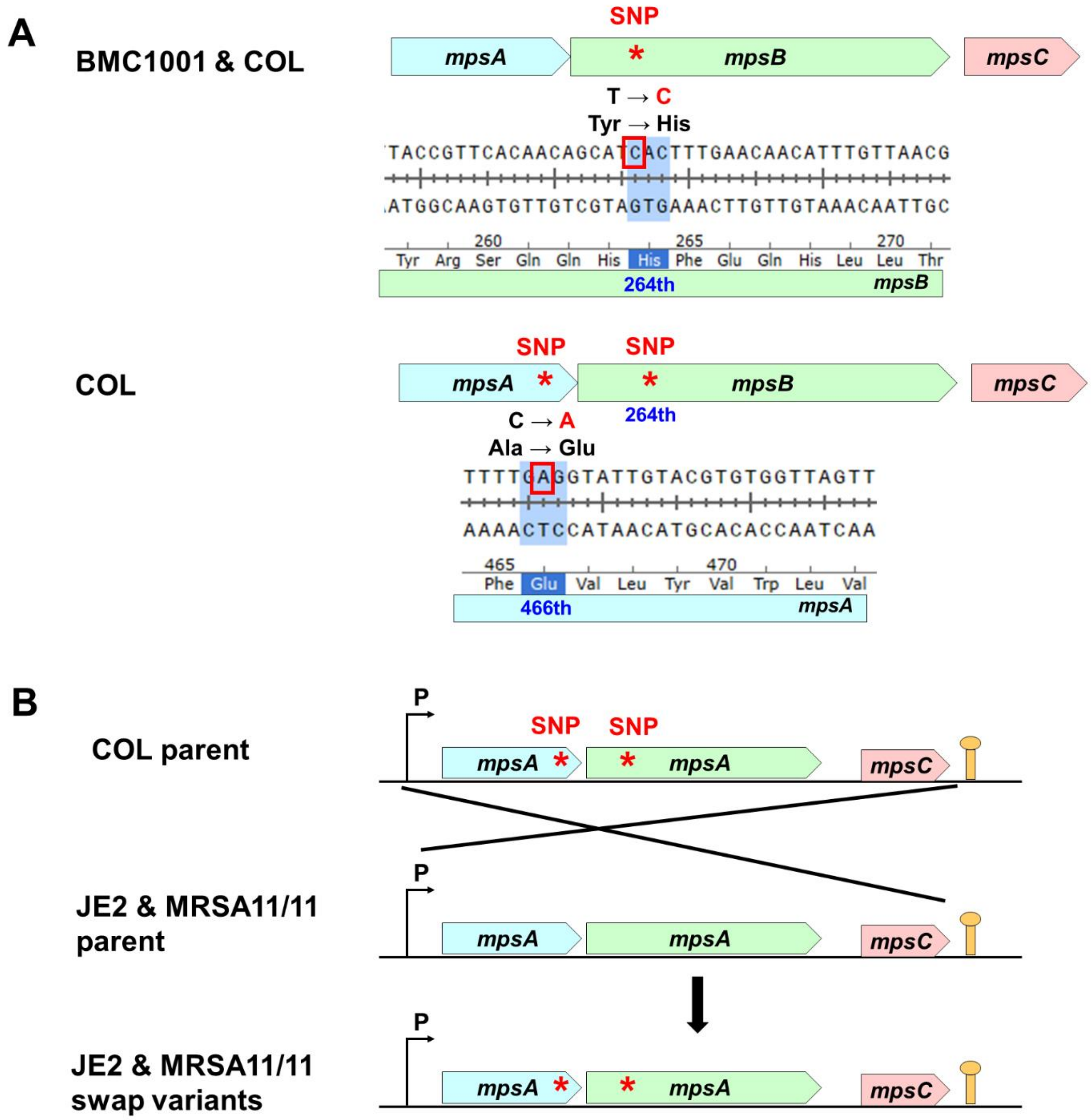

Supplementary Figure 1: (A) In both *S. aureus* BMC1001 and COL ($NaHCO_3$-nonresponsive MRSA strains), there is a nucleotide change or Single Nucleotide Polymorphism (SNP, marked with *) from T-to-C in the coding region of *mpsB*, causing a change in the 264th amino acid position of MpsB from tyrosine to histidine. In COL, there

is an additional nucleotide change, from C-to-A resulting in the change in the 466th amino acid of MpsA from alanine to glutamic acid. **(B)** The SNPs were introduced into the responsive strains by “swapping” the *mpsABC* region from COL to JE2 and MRSA 11/11 via recombination or two-point cross-over, generating JE2 and MRSA 11/11 swap variants which harbor both the SNPs.

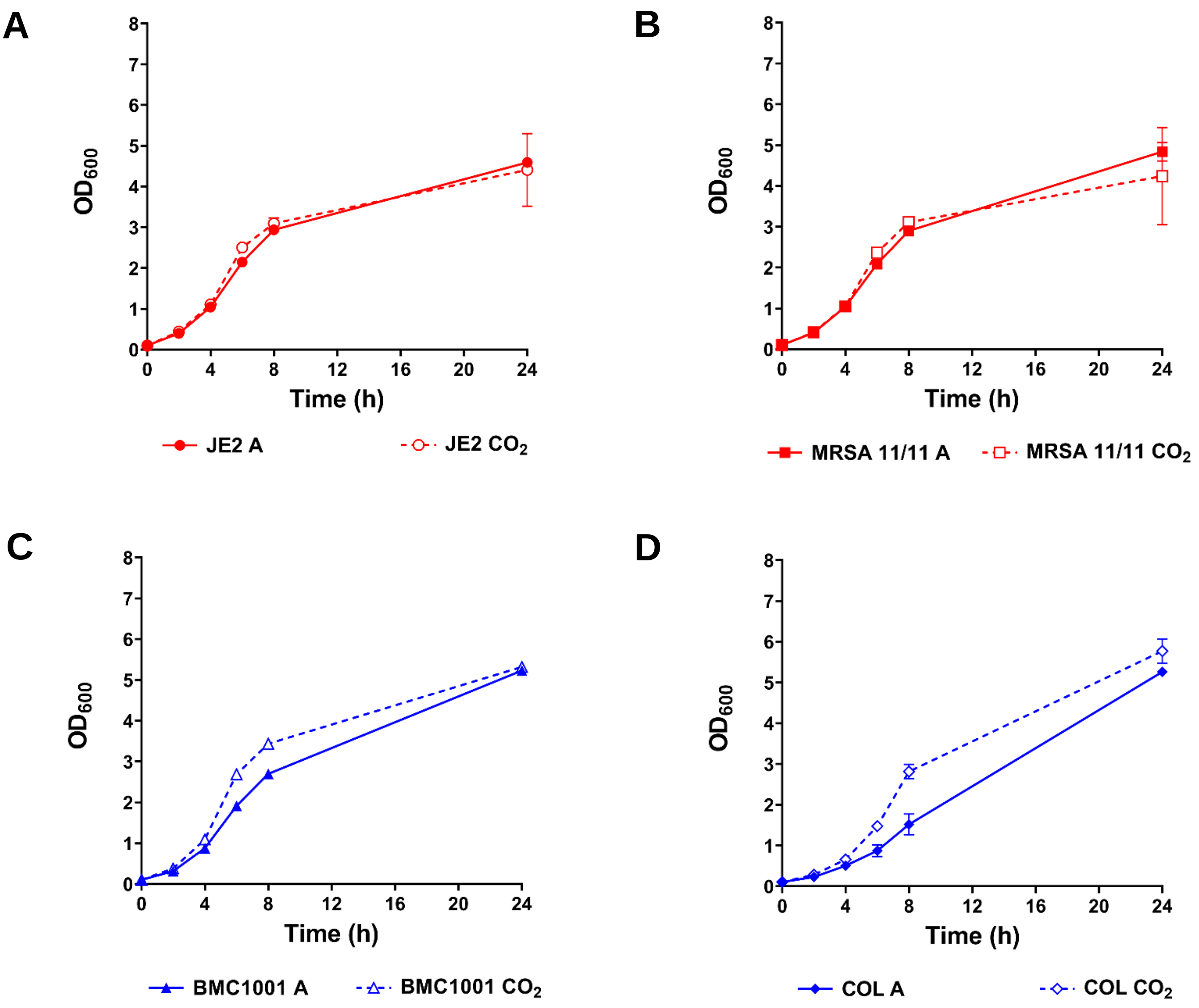

Supplementary Figure 2: Growth curves of *S. aureus* **(A)** JE2; **(B)** MRSA 11/11; **(C)** BMC 1001 and **(D)** COL in ambient air (A, solid lines) and 5% CO_2 conditions (CO_2, dashed lines). Strains were grown in cation-adjusted Mueller-Hinton Broth (CA-MHB) plus Tris. Data shown are mean ± standard deviation (SD) from two independent biological replicates.

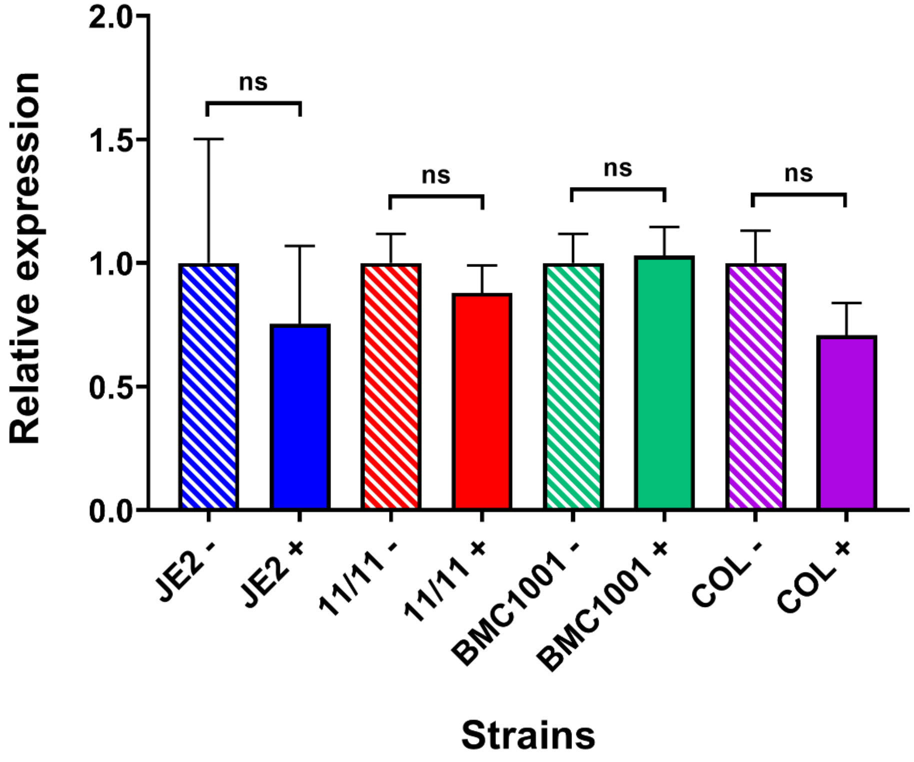

Supplementary Figure 3: Gene expression data in $NaHCO_3$-responsive (JE2 and MRSA11/11) and non-responsive strains (BMC1001 and COL) as a control to evaluate if oxacillin alone affects the expression of *mpsA*. Data were obtained by qRT-PCR of RNA from mid-exponential phase (3h) strains grown in cation-adjusted Mueller-Hinton Broth (CA-MHB) plus Tris with (+) or without (-) ½ MIC of oxacillin. 2% NaCl was included in growth media in which oxacillin was also included. For each strain, *mpsA* expression was normalized to the value obtained in CA-MHB-Tris (-), with this value set equal to 1.0. There was no significant difference (ns) between those grown with or without oxacillin for all the strain sets, as calculated by Student's *t* test. Data shown are mean ± standard deviation (SD) from two independent biological replicates, performed in duplicate for each strain/condition.

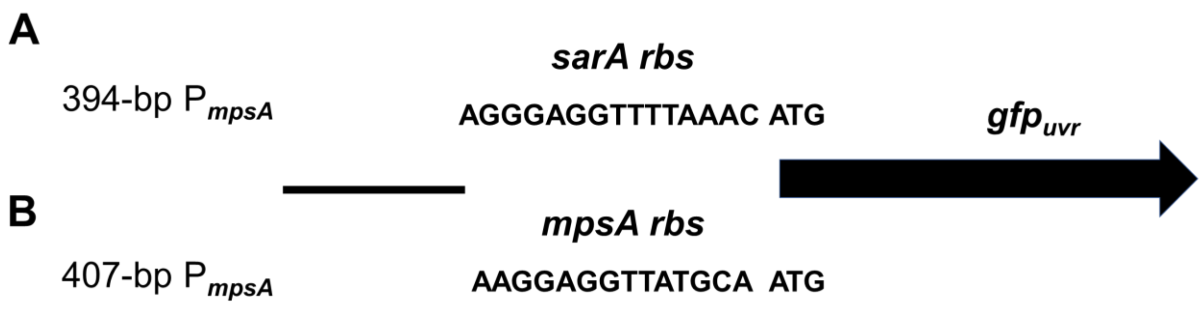

Supplementary Figure 4: Schematic of transcriptional and translational reporter fusions of *mpsA* promoter with **(A)** *sarA* RBS and (**B)** *mpsA* with its own RBS fused to gfp_{uvr} reporter gene.

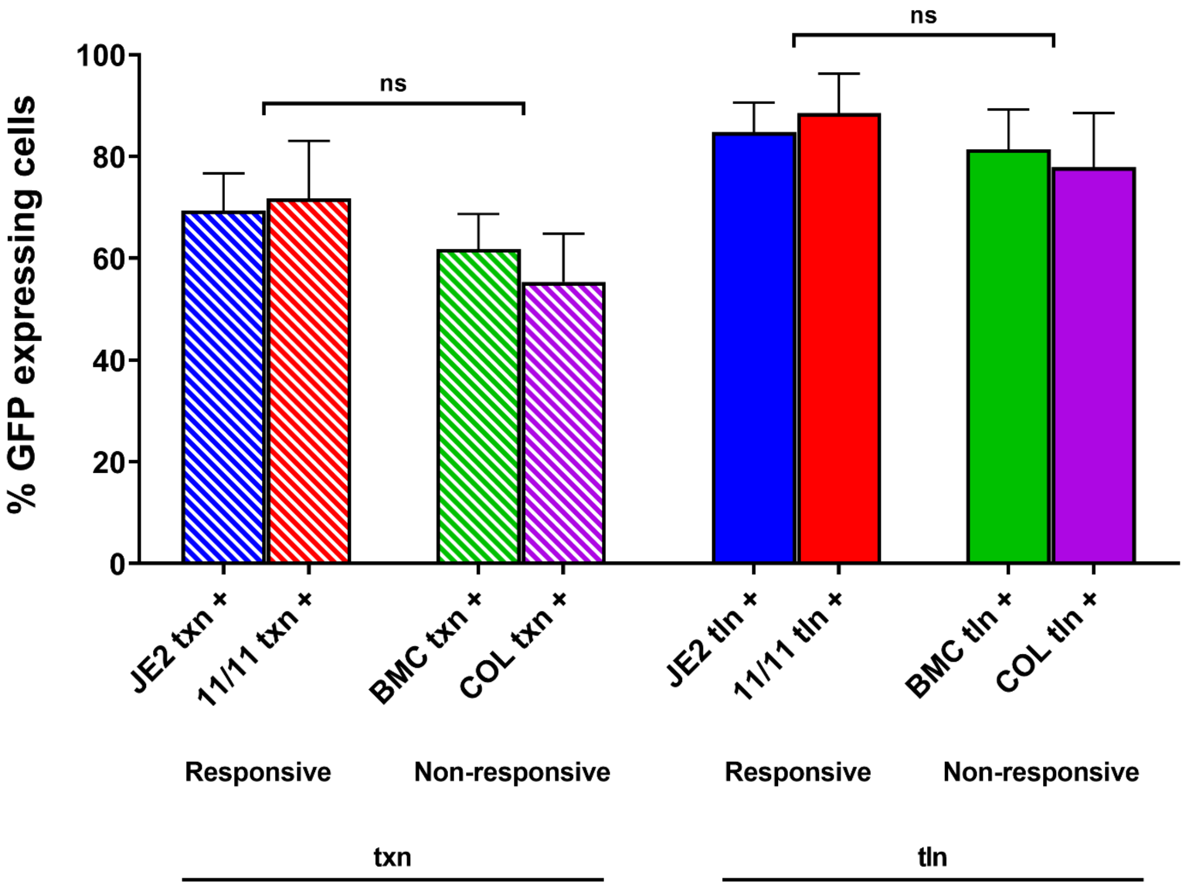

Supplementary Figure 5: Percentage of GFP expressing cells out of a total population of 10000 cells) following growth for 3 h in cation-adjusted Mueller-Hinton Broth (CA-MHB) plus Tris + 2% NaCl, ½ MIC oxacillin with $NaHCO_3$ (+). Average of GFP expressing cells (JE2, MRSA 11/11, BMC 1001, and COL) assessed by flow cytometry in transcriptional (txn) constructs (left) and in translational (tln) constructs (right). The constructs and conditions used are detailed in Materials & Methods. There was no significant difference (ns) in the percentage GFP expressing cells between the responsive (JE2 and MRSA 11/11) and non-responsive (BMC 1001 and COL) strains were determined by Student's *t* test). Data shown are the results of four independent biological replicates for each strain/condition.

A

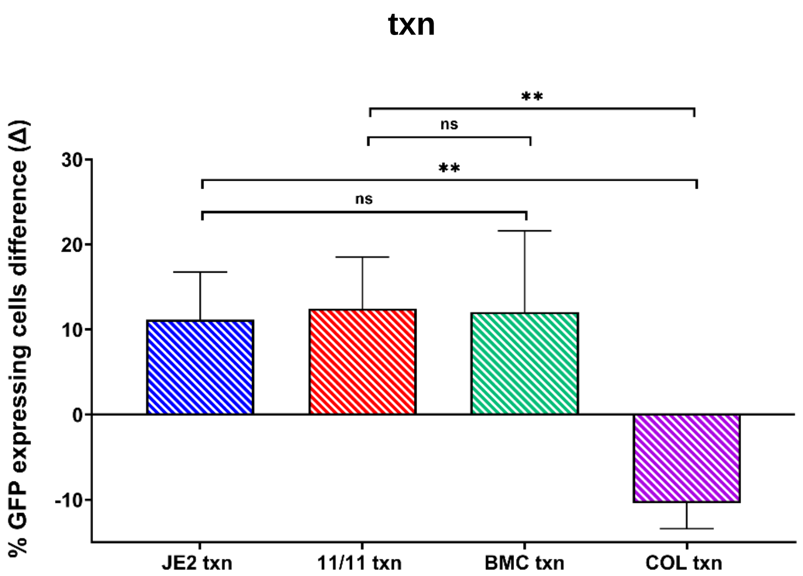

B

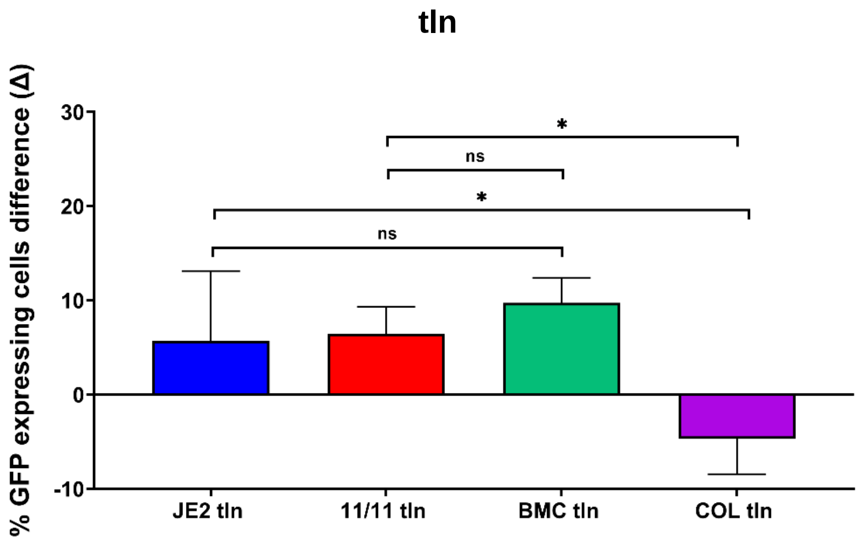

Supplementary Figure 6: Percentage of GFP expressing cells difference (Δ) of the strains JE2, MRSA 11/11, BMC1001 and COL grown with and without $NaHCO_3$ in the presence of ½ MIC oxacillin for **(A)** transcriptional (txn) and **(B)** translational (tln) constructs. The constructs and conditions used are detailed in Materials & Methods. Each bar shows the difference (increase/decrease) between the percentage of GFP expressing

cells of strain grown with and without $NaHCO_3$ (baseline levels) and the statistical significance between them were calculated using one-way ANOVA followed by Tukey's multiple comparison test. ($p > 0.05$, not significant, ns; $p < 0.05$ *; $p < 0.01$ **). Data shown are the results of four independent biological replicates for each strain/condition.

A

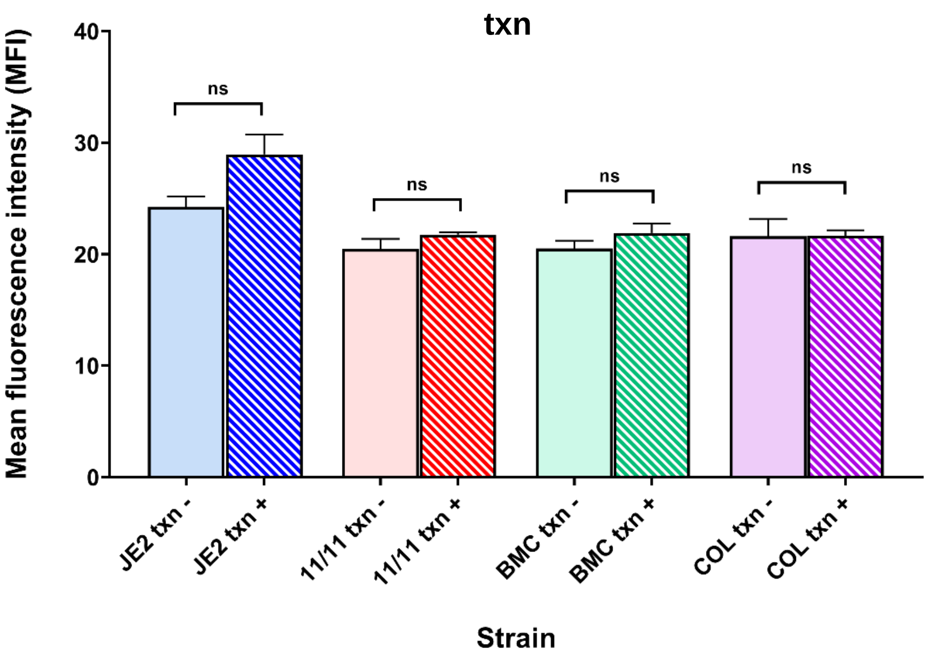

B

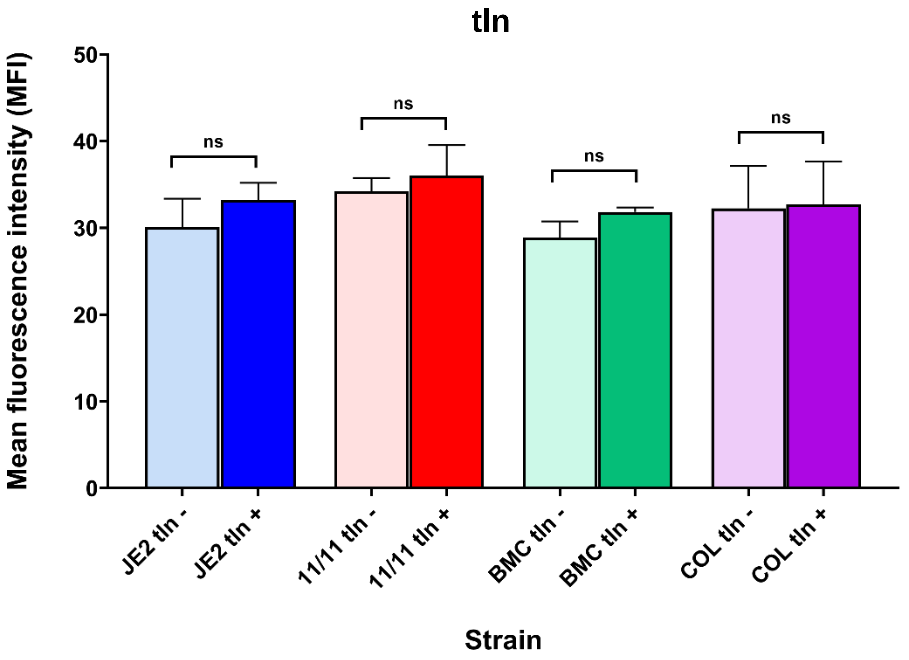

Supplementary Figure 7: Mean fluorescence intensity (MFI) out of a total population of 10000 cells) following growth for 3 h in cation-adjusted Mueller-Hinton Broth (CA-MHB) plus Tris with (+) or without (-) ½ MIC of oxacillin as a control to evaluate if oxacillin alone affects the transcriptional and translational efficiency of the promoter-GFP fusions

constructs. The constructs and conditions used are detailed in Materials & Methods. 2% NaCl was included in growth media in which oxacillin was also included. **(A)** MFI of the cells (JE2, MRSA 11/11, BMC 1001, and COL) as assessed by flow cytometry in transcriptional (txn) constructs and **(B)** in translational (tln) constructs. There was no significant difference (ns) in the MFI between those grown with or without oxacillin for both the txn and tln constructs, as determined by Student's *t* test. Data shown are mean ± standard deviation (SD) from two independent biological replicates, each with three technical replicates.

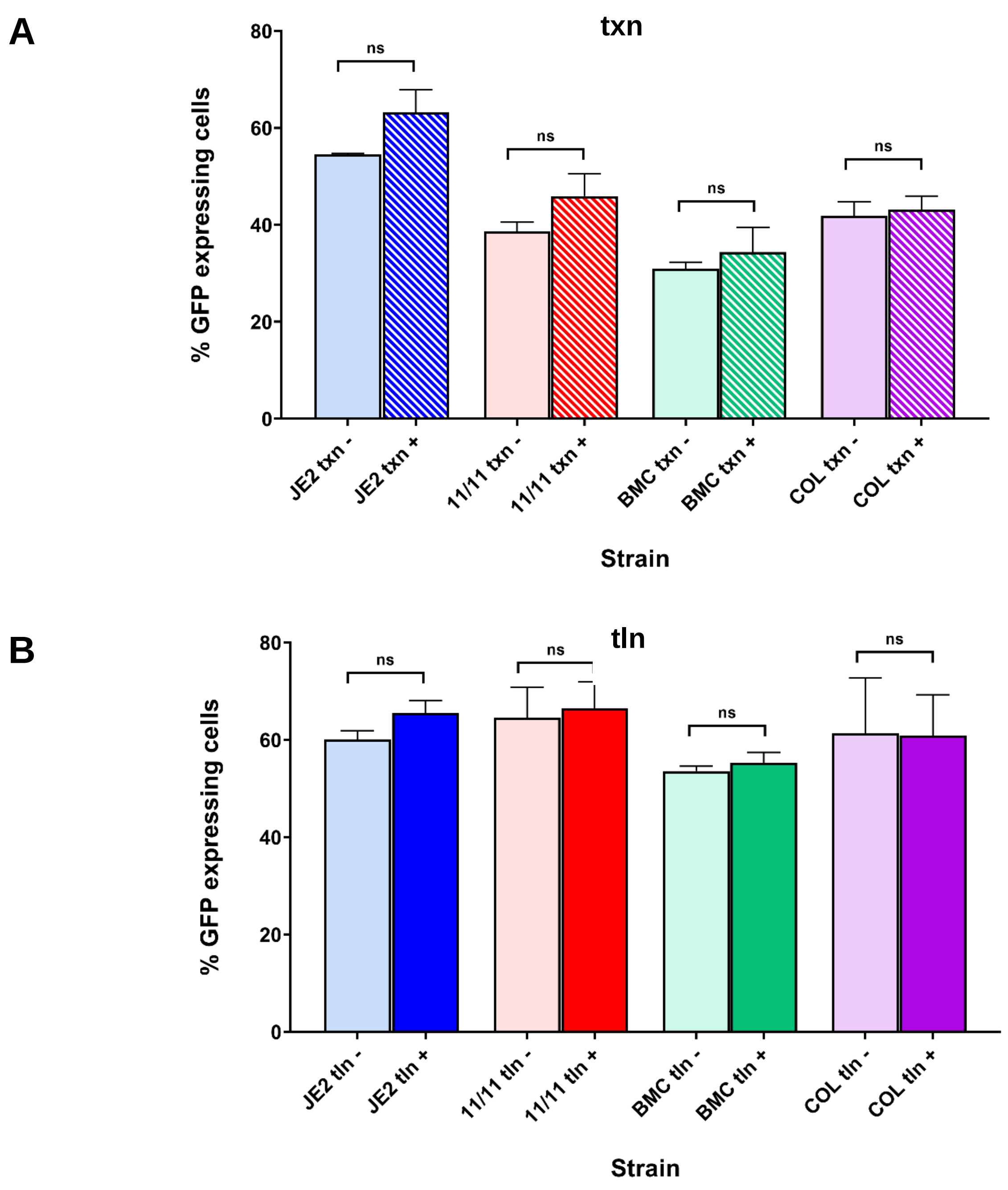

Supplementary Figure 8: Percentage of GFP expressing cells out of a total population of 10000 cells) following growth for 3 h in cation-adjusted Mueller-Hinton Broth (CA-MHB) plus Tris with (+) or without (-) ½ MIC of oxacillin as a control to evaluate if oxacillin alone affects the transcriptional and translational efficiency of the promoter-GFP fusions

constructs. 2% NaCl was included in growth media in which oxacillin was also included. The constructs and conditions used are detailed in Materials & Methods. **(A)** Average of GFP expressing cells (JE2, MRSA 11/11, BMC 1001, and COL) as assessed by flow cytometry in transcriptional (txn) constructs and **(B)** in translational constructs. There was no significant difference (ns) in the percentage of GFP expressing cells between those grown with or without oxacillin for both the txn and tln constructs, as determined by Student's *t* test. Data shown are mean ± standard deviation (SD) from two independent biological replicates, each with three technical replicates.

SUPPLEMENTARY TABLES

Supplementary Table 1: Minimum inhibitory concentrations (MICs) of oxacillin in JE2 and MRSA11/11 swap variants

	Oxacillin (µg/mL)	
	Ambient air	
**Strains**	**CA-MHB Tris**	**CA-MHB Tris + 44 mM $NaHCO_3$**
**Swap variants**		
JE2 swap variant	64	2
MRSA 11/11 swap variant	64	2
**Responsive**		
JE2 parent	64	1
MRSA 11/11 parent	32	0.5
**Non-responsive**		
COL parent	256	512

Supplementary Table 2: Bacterial strains used in the study

Strains	Descriptions	Reference
Staphylococcus aureus		
**For the construction of *mpsABC* deletion mutants**		
S. aureus JE2 (parent)	*S. aureus* USA 300 FPR375 derivative, cured of three plasmids	(1)
S. aureus JE2 Δ*mpsABC*	*S. aureus* JE2 Δ*mpsABC* (deletion of SAUSA300_0425, SAUSA300_0426 and SAUSA300_0426)	(2)
S. aureus JE2 Δ*mpsABC* (pRB473 *mpsABC*)	*S. aureus* JE2 Δ*mpsABC* complemented with *mpsABC*	(2)
MRSA 11/11 (parent)	Daptomycin non-susceptible methicillin-resistant *S. aureus* USA 300 isolate	(3)
MRSA 11/11 Δ*mpsABC*	MRSA 11/11 Δ*mpsABC* (deletion of *mpsA, mpsB* and *mpsC*)	This study
MRSA 11/11 Δ*mpsABC* (pRB473 *mpsABC*)	MRSA 11/11 Δ*mpsABC* complemented with *mpsABC*	This study
BMC1001 (parent)	Clinical bloodstream isolate	(4)
BMC1001 Δ*mpsABC*	*S. aureus* Δ*mpsABC* (deletion of *mpsA, mpsB* and *mpsC*)	This study

BMC1001 Δ*mpsABC* (pRB473 *mpsABC*)	BMC1001 Δ*mpsABC* complemented with *mpsABC*	This study
COL (parent)	A representative member of an early MRSA lineage first identified in the 1960s	(5)
COL Δ*mpsABC*	*S. aureus* COL Δ*mpsABC* (deletion of SACOL0494, SACOL0494 and SACOL0494)	This study
COL Δ*mpsABC* (pRB473 *mpsABC*)	*S. aureus* COL Δ*mpsABC* complemented with *mpsABC*	This study
**For the construction of GFP reporter strains**		
Plasmid pALC1484	Vector for promoterless gfp reporter gene (gfp_{uvr}, $chlor^R$)	(6)
ALC9475	JE2 (pALC1484 with 394-bp promoter region of the *mpsA* gene with the *sarA* ribosome binding site before *gfp* gene).	This study
ALC9476	MRSA11/11 (pALC1484 with 394-bp promoter region of *mpsA* gene with the *sarA* ribosome binding site before *gfp* gene).	This study
ALC9477	BMC1001 (pALC1484 with 394-bp promoter region of *mpsA* gene with the *sarA* ribosome binding site before *gfp* gene).	This study

ALC9478	COL (pALC1484 with 394-bp promoter region of *mpsA* gene with the *sarA* ribosome binding site before *gfp* gene).	This study
ALC9410	BMC1001 with the empty plasmid (pALC1484)	This study
ALC9414	MRSA11/11 with the empty plasmid (pALC1484)	This study
ALC9418	COL with the empty plasmid (pALC1484)	This study
ALC9485	JE2 with the empty plasmid (pALC1484)	This study
ALC9479	JE2 (pALC1484 with 407-bp *mpsA* promoter region with *mpsA* ribosome binding site before *gfp* gene).	This study
ALC9480	MRSA 11/11 (pALC1484 with 407-bp *mpsA* promoter region with *mpsA* ribosome binding site before *gfp* gene).	This study
ALC9481	BMC1001 (pALC1484 with 407-bp *mpsA* promoter region with *mpsA* ribosome binding site before *gfp* gene).	This study
ALC9482	COL (pALC1484 with 407-bp *mpsA* promoter region with *mpsA* ribosome binding site before *gfp* gene).	This study
**For the construction of SNP swap variants**		
ALC9556	JE2 with COL *mpsAB* region swap variant	This study
ALC9557	MRSA 11/11 with COL *mpsAB* region swap variant	This study

Supplementary Table 3: Oligonucleotides used in this study

Primer name	Sequence (5’→3’)
**For the construction of *mpsABC* deletion mutants**	
Upstream mpsABC Fwd	AATTCCGGAGCTCGGTACCCTCCATGCTAAAAGGTTCAATG
Upstream mpsABC rev	TATCCTCAAGCTAATCTCTCGCATAATTGC
Downstream mpsABC fwd	GAGAGATTAGCTTGAGGATAATTTGGAAAAG
Downstream mpsABC fwd	ACAGATCTGCGCGCTAGCCCTGTGCACTTGTTTCATAG
**For the construction of reporter strains**	
mpsA EcoR1	AAGA AGA GAA TTC AAA AGG ACG GGA AAT ACT GCC TAA
mpsA XbaI	AGG AGAA TCT AGA TAAT CTC TCG CAT AAT TGC TTA TGT ATA
mpsA rbs F	AGGAGAATCTAGAAGGAGGTTATGCAATGATTAAAGGAGAAG AACTTTTCACT
1484 *EcoRI*	AGA GGA TCC CCG GGT ACC GA
**For qRT-PCR**	
mpsA Fwd	CACGTGAGTCTGCGAAATTA
mpsA Rev	CCGGTATCATGACAGCTAATAC
gyrB Fwd	CGCAGGCGATTTTACCATTA
gyrB Rev	GCTTTCGCTAGATCAAAGTCG

**For the construction of SNP swap variant**	
mpsAB F	AAG AGA GGA TCC ATG AGT TAA CTT CAT TGT ACA TAG TTA
mpsAB R	AAG AGA GGA TCC GAA GCT AGA ACA TTG TAG ATA TGA TGA
mpsAB Verify F	GAG TTG GAC AAT GCC GAA GCG TGA

Supplementary references

1. Fey PD, Endres JL, Yajjala VK, Widhelm TJ, Boissy RJ, Bose JL, Bayles KW. 2013. A genetic resource for rapid and comprehensive phenotype screening of nonessential *Staphylococcus aureus* genes. mBio 4:e00537-12.
2. Fan SH, Liberini E, Gotz F. 2021. *Staphylococcus aureus* Genomes Harbor Only MpsAB-Like Bicarbonate Transporter but Not Carbonic Anhydrase as Dissolved Inorganic Carbon Supply System. Microbiol Spectr 9:e0097021.
3. Murthy MH, Olson ME, Wickert RW, Fey PD, Jalali Z. 2008. Daptomycin non-susceptible meticillin-resistant *Staphylococcus aureus* USA 300 isolate. J Med Microbiol 57:1036-1038.
4. Yang SJ, Xiong YQ, Boyle-Vavra S, Daum R, Jones T, Bayer AS. 2010. Daptomycin-oxacillin combinations in treatment of experimental endocarditis caused by daptomycin-nonsusceptible strains of methicillin-resistant *Staphylococcus aureus* with evolving oxacillin susceptibility (the "seesaw effect"). Antimicrob Agents Chemother 54:3161-9.
5. Dyke KG. 1969. Penicillinase production and intrinsic resistance to penicillins in methicillin-resistant cultures of *Staphylococcus aureus*. J Med Microbiol 2:261-78.
6. Kahl BC, Goulian M, van Wamel W, Herrmann M, Simon SM, Kaplan G, Peters G, Cheung AL. 2000. *Staphylococcus aureus* RN6390 replicates and induces apoptosis in a pulmonary epithelial cell line. Infect Immun 68:5385-92.
